# Diagnosis and treatment of acute inflammatory sacroiliitis in pregnant or post-partum women: a systematic review of the current literature

**DOI:** 10.1007/s12306-023-00786-x

**Published:** 2023-06-20

**Authors:** G. Viroli, T. Cerasoli, F. Barile, M. Modeo, M. Manzetti, M. Traversari, A. Ruffilli, C. Faldini

**Affiliations:** IRCCS Istituto Ortopedico Rizzoli, 1st Orthopaedics and Traumatology Clinic, University of Bologna, Bologna, Italy

**Keywords:** Sacroiliitis, Sacroiliac joint injection, SIJ conservative therapy, Pregnancy, Sacroiliitis in pregnancy

## Abstract

The aim of the present study is to systematically review the current literature about diagnosis and treatment of acute inflammatory sacroiliitis in pregnant or post-partum women. A systematic search was carried out according to the Preferred Reporting Items for Systematic Reviews and Meta-Analyses guidelines. Data about clinical presentation, diagnosis methods and treatment strategies were retrieved from included studies and reported in a table. After screening, five studies on 34 women were included; they were all affected by acute inflammatory sacroiliitis. Clinical examination and magnetic resonance imaging were used to confirm diagnosis. In four studies, patients were treated with ultrasound-guided sacroiliac injections of steroids and local anesthetics, while one study used only manual mobilization. Clinical scores improved in all patients. Ultrasound-guided injections proved to be a safe and effective strategy for inflammatory sacroiliitis treatment during pregnancy or post-partum.

## Introduction

The extensive physiologic and anatomic changes that occur during pregnancy pose difficult challenging questions to the clinicians. Strikingly, up to 25% of pregnant women report temporary disabling musculoskeletal symptoms, and roughly one-third report impairment of daily activities related to pain [1].

Prior retrospective reviews demonstrated incidence rates around 50% of lumbopelvic and sacroiliac pain during pregnancy; moreover, a considerable number of these patients have pain persisting as long as 2 years post-partum [2, 3].

The sacroiliac joint (SIJ) is a diarthrodial joint that connects the lumbosacral spine to the pelvis; it is the largest axial joint of the human body and is considered a synovial joint with a fibrous capsule containing synovial fluid [4]. It transmits forces to lower limbs and absorbs ascending forces: infact, SIJ is a triplanar shock absorber which dissipates axial compression and rotational stresses and is more resistant to lateral forces than the lumbar spine [5]. It has minimal movement, between 2.5 degrees of rotation and 0.7 mm of translation; nevertheless, the innervation is highly represented and can trigger a lot of pain [6, 7].

During pregnancy, the sacroiliac joint has to endure extra burden due to biomechanical changes, leading to pubic instability, inflammation, bone edema and stress fractures; moreover, the release of the pregnancy-related hormone relaxine allows for pelvic expansion and increased movement [8, 9].

There is a consensus regarding the inadequacy of treatments for pelvic, lumbar and SIJ pain during pregnancy. A major and somehow justified therapeutic limitation involves the concern over the adverse effects of drugs and treatment strategies on the mother and developing fetus. Moreover, pain is commonly perceived as a natural element of pregnancy. Therefore, therapeutic strategy is often based on prevention, and conservative choices are more frequently preferred. Nevertheless, some studies have been published on sacroiliac pain management in pregnant women, through steroid and/or local anesthetics injections and/or through manual therapy.

The aim of the present study is to review the current literature on the topic, in order to help physicians managing this kind on symptoms on pregnant women.

## Materials and methods

A systematic review of the available English literature on three large electronic databases (Scopus, Embase and Pubmed) was performed in February 2023. The search strategy was based on a combination of the following keywords: “sacroiliac,” “sacroiliac,” “sacroiliitis,” “aseptic,” “pregnancy,” “pregnant,” “post-partum,” “inject*” “conservative.”

No limits regarding the publication year were applied. Additional articles have been found through a cross-reference search of the eligible studies. Two authors (TC and MM) independently screened all potentially relevant titles and abstracts, and any disagreement was solved by the senior authors (AR and CF). The search was limited to human data in pregnant and post-partum women. The Preferred Reporting Items for Systematic reviews and Meta-Analyses recommendations were followed during the preparation of this review [10]. Randomized controlled trials (RCTs), retrospective or prospective observational studies, case series or reports, who met the PICO (population, intervention, comparison and outcomes) were considered; manuscript about septic sacroiliitis was excluded. The outcome of interest was looking at the diagnostic and therapeutic management of aseptic sacroiliitis in pregnant women and to determine what interventions have an impact on patient-reported pain scores and functional ability.

Two authors (TC and MM) independently assessed the quality of included trials using the NIH tool [11].

Studies on pregnant and post-partum patients reporting on one or more of our outcomes of interest were included in this review. Reference data, populations and outcomes were extracted from the articles into pre-specified tables using a standardized data extraction procedure by two of the authors (MM and MT). They extracted information on studies’ general characteristics (including design, number of arms and primary outcomes), participants (population and sample size), interventions (diagnostic modality or therapeutic technique), comparator (if any), parameters used for assessing efficacy of the intervention and summary of main outcomes.

## Results

### Baseline studies characteristics and quality assessment

**Fig. 1:**
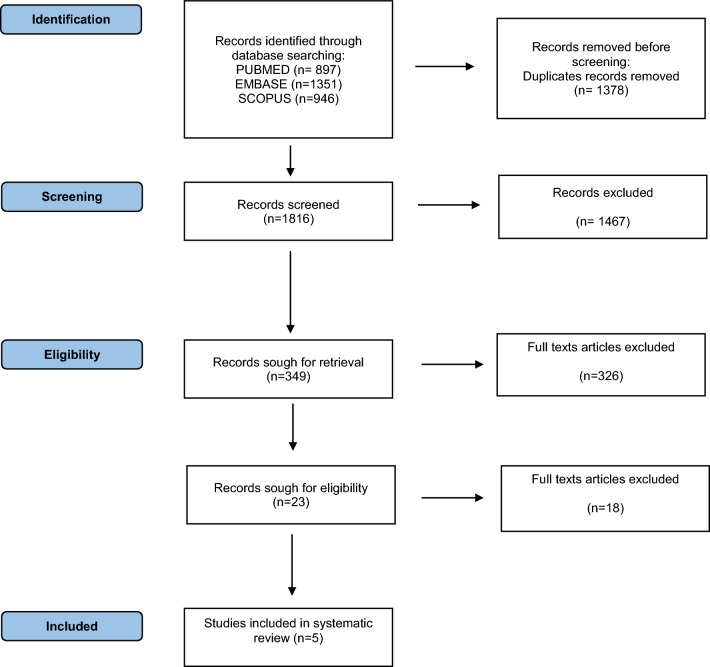
PRISMA flowchart

The search result and study selection flowchart are reported in Fig. 1. A total of 3194 records were identified through database searching. After excluding duplicates and screening titles and abstract, 1816 studies were found to be relevant to the objectives of this review, and the full texts were retrieved. At the end of the full-text screening, five articles were included for qualitative analysis: two retrospective cohort studies [12, 13] and three case reports [14–16].

The methodological quality of the two retrospective studies was assessed and is reported in Table 1. All included studies and their most relevant results are summarized in Table 2.

**Table 1:** NIH scale quality assessment for retrospective studies

Studies	Was the study question or objective clearly stated?	Was the study population clearly and fully described, including a case definition?	Were the cases consecutive?	Were the subjects comparable?	Was the intervention clearly described?	Were the outcome measures clearly defined, valid, reliable and implemented consistently across all study participants?	Was the length of follow-up adequate?	Were the statistical methods well-described?	Were the results well-described?	Quality summary
Sevas Comlek, 2020 [12]	**✔**	**✔**	**✔**	**✔**	**✔**	**✔**	**✗**	**✗**	**✔**	**2**
Xiaoxu Lu, 2022 [13]	**✔**	**✔**	**✔**	**✔**	**✔**	**✔**	**✗**	**✗**	**✔**	**2**

Quality was rated as 0 for poor (0–3 out of 9 questions),* 1* for fair (4–6 out of 9 questions) *or* 2 for good (7–9 out of 9 questions); *NA* not applicable and *NR* not reported [11]

**Table 2:** Included studies and relevant results

Author	Design	LoE	Patients n	Mean age	Signs and symptoms	Diagnosis	Treatment	Outcome
Xiaoxu Lu, 2022 [13]	RS	IV	11	30	Hip and leg pain, ESR augmented in all patients, CRP in 5/11	MRI	NSAIDs, betamethasone + ropivacaine + methylcobalamin SIJ injection (US or fluoroscopy guided), myofascial trigger points release	After 2–4 injections: VAS significantly lowered, provocative tests -, ESR and CRP normal
Ferias de-Aragão, 2021 [14]	CR	VI	1	42	LBP	US	Ropivacaine + methylprednisolone SIJ injection (US guided)	Complete improvement of pain
Savas Comlek, 2020 [12]	RS	IV	20	31.0 ± 2.9	LBP	MRI	Methylprednisolone + lidocaine SIJ injection (US guided)	Significant ODI and VAS reduction
Vincent, 2019 [15]	CR	VI	1	30	LBP	US	Lidocaine + triamcinolone acetonide SIJ injection	After procedure, VAS 0/10; after 14 days, VAS 2/10
Paulsen Te, 1993 [16]	CR	VI	1	32	SIJ pain	n.r	Manual mobilization	The patient reported to be “very happy” after five treatments

*LoE* level of evidence, *N* number, *RS* retrospective study, *CR* case report, *MRI* magnetic resonance imaging, *US* ultrasound, *CT* computed tomography, *N.r* not reported, *SIJ* sacroiliac joint, *LBP* low back pain, *ESR* erythrocyte sedimentation rate, *CRP* c-reactive protein, *VAS* visual analog scale and *ODI* Oswestry disability index

### Population

A total of 34 pregnant or post-partum women affected by LBP and/or sacroiliac pain nonresponsive to conservative management were included. Mean age ranged between 30 and 42 years.

All patients reported intense pain, with visual analog scale (VAS) > 5 at resting state and aggravated (VAS > 7) by standing, walking and sitting [13]. According to all authors, the intensity of the pain of all their patients was exacerbated by side-to-side compression of the pelvis; the Patrick’s test and the straight leg raise test were positive and direct palpation of the sacroiliac joint produced severe pain. No author reported any motor or sensory impairment. The strength of pelvic girdle muscles was diminished because of pain; the bladder and bowel functions of all patients were normal.

### Interventions

All but one patient [16] received ultrasound-guided corticosteroids sacroiliac joint injections. The injected corticosteroid was betamethasone in one study (11 patients) [13], methylprednisolone in two studies (21 patients) [14], 12] and triamcinolone acetonide in one study (1 patient) [15]. Two authors performed manual therapy: Xiaoxu et al. [13] performed myofascial trigger points release on their 11 patients, while Paulsen Te et al. [16] performed manual mobilization of the sacroiliac joint.

### Outcomes

All studies reported good outcomes. Only three authors [12, 13, 15] reported pain scores at follow-up. They all reported a significant reduction of VAS and/or ODI. Two authors [14, 16] did not use objective scores; nevertheless, they both reported a good satisfaction of their patients.

## Discussion

The incidence of lumbopelvic pain during gestation has been reported to be around 50% [15]. The severity and earlier development of pain during pregnancy has been linked to the advancement toward chronic disabling post-partum pain [17]. Therefore, prompt diagnosis and treatment are extremely important to avoid developing chronic pain and long-term limitations to daily life [14].

As the largest true synovial joint in the body and a highly specialized diarthrodial between the surface of the ilium and the sacrum, the sacroiliac joint supports weight, maintains stability and enhances joint strength by absorbing and transferring forces; it is a strong ligament and muscle network system that connects the spine to the pelvis [18]. Nevertheless, its range of motion is limited. Several factors contribute to its inflammation during pregnancy or post-partum. Mechanical and physiological changes in pregnant women alter the activity of the SIJ, leading to sacroiliitis: the high estrogen and progesterone augment ligament relaxation, promoting pubic symphysis separation; SIJ surface injury, maternal weight increase, uterine contraction and pelvic floor muscle tension increase, are all factors that aggravate sacroiliac joint pressure and promote sacroiliitis development [19, 20, 21].

The aim of the present review was to systematically analyze the existing literature about inflammatory sacroiliitis diagnosis and management during pregnancy or post-partum. According to our results, SIJ steroid injections represent a safe and effective option. All included studies reported good clinical outcomes, with VAS and/or ODI improvement.

The non-specific symptoms and signs, various characteristics of acute sacroiliac pain, result in the diagnosis being complicated. Acute sacroiliitis is characterized by lumbosacral pain accompanied by hip pain, proximal thigh pain and groin pain. It results in significant restraint in the activity of the sacroiliac joint, which seriously damages the health and quality of life [13]. The reference diagnostic standard for sacroiliac joint pain, recommended by the International Association for the Study of Pain in 1994, is as follows: 1) pain in the region of the SIJ; 2) pain is reproduced by stressing the SIJ with clinical tests and 3) selectively infiltrating the symptomatic joint completely relieves the symptoms [22]. Physical examination provocative tests such as Gaenslen test and Patrick’s test are extremely helpful for diagnosis; nevertheless, MRI is considered the gold standard [13]. Infact, MRI detects inflammatory changes before the structural change occurs, and accurately distinguishes between infective and inflammatory etiologies, without exposing the pregnant women to ionizing radiation [13].

The mainstays of initial treatment of sacroiliitis are divided into nonintervention and interventional treatment. The mainstays of noninterventional therapy include rest, massages, physical therapy and rehabilitation exercise, associated (after delivery) to anti-inflammatory drugs (NSAIDS). However, anatomical changes related to pregnancy may limit physical therapy modalities, while recognized fetal risks linked to nonsteroidal anti-inflammatory drugs narrow their use. Therefore, interventional therapy is the key, including acupuncture, ozone therapy and ultrasound-guided injections [15]. There is moderate-level evidence for the efficacy of image-guided sacroiliac joint injections with local anesthetic and steroid for relieving pain in sacroiliac instability [12]; image-guided injections are the preferred method to achieve safe and precise intra-articular needle placement, and the latest research indicates that ultrasonography provides the same success rate as fluoroscopy, without ionizing radiations exposure [23]. Remission rates lasting 1–6 months have been reported in 60%–80% of the patients [24].

Intra-articular injection of anesthetics can provide significant analgesia: a long-acting agent like bupivacaine is preferred for the advantage of prolonging analgesia duration; corticosteroids have strong anti-inflammatory, anti-allergic and immunosuppressive functions, and can effectively relieve the pain [13].

In conclusion, clinical examination and magnetic resonance are the best diagnostic tools for acute sacroiliitis in pregnant and post-partum women, and ultrasound-guided sacroiliac joint injections of steroids in association with local anesthetics (usually bethamethasone + ropivacaine) are a safe and effective treatment strategy, providing a very good relief of symptoms, without risks for the fetus or the newborn.
